# Dilatation of the bridging cerebral cortical veins in childhood hydrocephalus suggests a malfunction of venous impedance pumping

**DOI:** 10.1038/s41598-022-17465-9

**Published:** 2022-07-29

**Authors:** Grant A. Bateman, Alexander R. Bateman, Gopinath M. Subramanian

**Affiliations:** 1 Department of Medical Imaging, John Hunter Hospital, Locked Bag 1, Newcastle Region Mail Center, Newcastle, NSW 2310 Australia; 2 Newcastle University Faculty of Health, Callaghan Campus, Newcastle, NSW Australia; 3 School of Mechanical Engineering, University of New South Wales, Sydney, NSW Australia; 4 Department of Paediatric Neurology, John Hunter Hospital, Newcastle, NSW Australia

**Keywords:** Biomedical engineering, Experimental models of disease

## Abstract

Dogs with a naturally occurring form of hydrocephalus have an elevated transmural venous pressure leading to cortical vein dilatation. The purpose of this study is to discover if there is vein dilatation in childhood hydrocephalus and to estimate the pressure required to maintain any enlargement found. Children with hydrocephalus between the ages of 4 and 15 years were compared with a control group. Magnetic resonance venography (MRV) and flow quantification were performed. The arterial inflow, sagittal sinus and straight sinus venous outflow were measured and the outflow percentages compared to the inflow were calculated. The cross-sectional area of the veins were measured. There were a total of 18 children with hydrocephalus, compared to 72 age and sex matched control MRV’s and 22 control flow quantification studies. In hydrocephalus, the sagittal sinus venous return was reduced by 12.9%, but the straight sinus flow was not significantly different. The superficial territory veins were 22% larger than the controls but the vein of Galen was unchanged. There is evidence of a significant increase in the superficial vein transmural pressure in childhood hydrocephalus estimated to be approximately 4 mmHg. An impedance pump model is suggested to explain these findings.

## Introduction

In hydrocephalus, there is deformation of the brain parenchyma. In engineering terms such a deformation requires the expenditure of energy. In a hydraulic system, energy expenditure requires a pressure differential^1^. The pressure difference deforming the brain in hydrocephalus has remained elusive. In a study of adult hydrocephalus, a prediction of an elevation in superior sagittal sinus (SSS) pressure of 3–4 mmHg above normal was made based on the evidence of increased collateral flow bypassing the sinuses^2^. It was hypothesised that collapse of the sagittal sinus would set up a pressure differential between the superficial and deep brain parenchyma and account for the ventricular dilatation found^1^. However, when this differential was estimated using Poiseuille’s equation, it was less than predicted being only 1.2 mmHg^1^.

Another problem with the sinus pressure differential hypothesis arises because of the subarachnoid course of the bridging cortical veins (BCV) and the vein of Galen. The capillary and venular pressure in the brain is thought to be maintained by a Starling resistor mechanism because the veins pass through the subarachnoid space^3^. The cortical veins are a series of collapsible tubes in which the pressure external to the tubes (CSF pressure) usually exceeds the outflow pressure (sinus pressure) therefore the perfusion pressure will only depend on the inflow pressure and the CSF pressure. If the CSF pressure is increased, the pressure in the cortical veins must be slightly higher for blood to flow. Thus, the pressure across the cortical vein wall (transmural pressure) is expected to remain very nearly constant^4^. Therefore, a pressure difference in the sinuses would be irrelevant to the brain parenchyma if both brain territories had their pressures set by the intracranial pressure^1^ and not the outflow sinus pressure.

**Figure 1:**
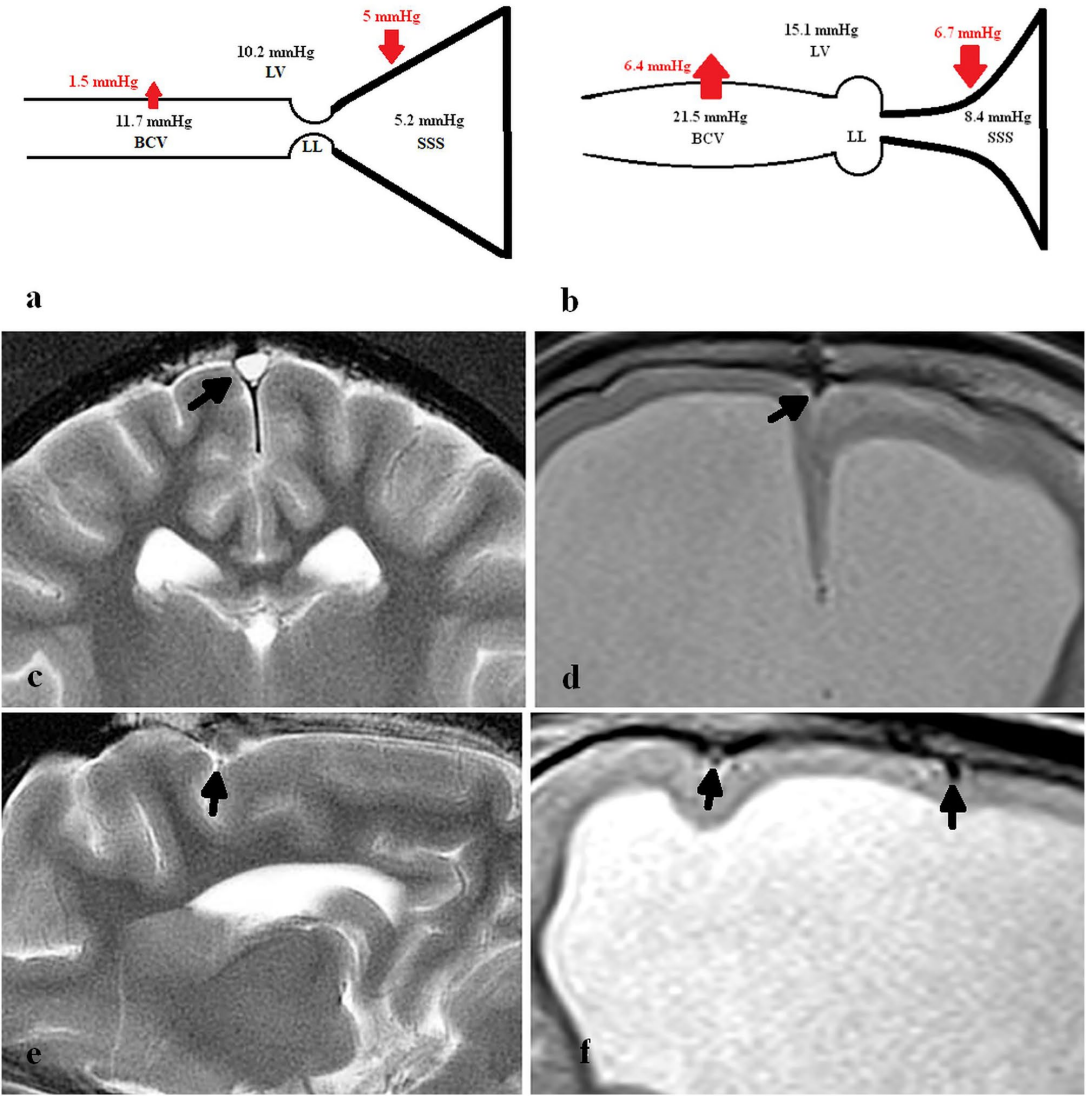
Pressure changes in the naturally occurring hydrocephalus in dogs. (**a**) A diagram of the bridging cortical vein (BCV), lateral lacunae (LL), superior sagittal sinus (SSS) and lateral ventricle (LV) with pressures in normal dogs appended from Portnoy et al.^5^. The pressure differences across vein walls are shown in red with arrows. (**b**) A diagram of the pressures found in dogs with a naturally occurring form of hydrocephalus from Portnoy et al.^5^. Note the pressure across the sagittal sinus wall is increased by 1.7 mmHg and the vein is compressed. The pressure across the BCV is increased by 327% and the vein is depicted as dilated. (**c**) A coronal T2 image of a normal dog with the arrow indicating the superior sagittal sinus. (**d**) A coronal T2 image of a naturally hydrocephalic dog showing the sagittal sinus to be much smaller than in 1c. (**e**) A sagittal T2 slice, just off centre, in a normal dog showing a BCV as a black dot at the arrow head. (**f**) A sagittal slice from the hydrocephalic dog showing significantly dilated BCVs (arrows).

However, the Starling resistor model may be incomplete in hydrocephalus. Comparing pressures in normal dogs to those with a naturally occurring form of hydrocephalus, the lateral ventricle, cortical vein and sagittal sinus pressures in the controls were 10.2, 11.7 and 5.2 mmHg respectively, with hydrocephalic dogs being 15.1, 21.5 and 8.4 mmHg respectively^5^ (see Fig. 1a,b). Note the sagittal sinus pressure is mildly increased by 3.2 mmHg but the BCV pressure is increased by 9.8 mmHg in hydrocephalus compared to controls. The increase in sinus pressure in hydrocephalic dogs can be explained by venous collapse. Modelling of hydrocephalus in dogs shows the entire sinus length to be narrowed^6^ (see Fig. 1c,d). In dogs, between the cortical veins and the superior sagittal sinus, lie the lateral lacunae, which are small venous lakes between the leaves of dura which are thought to act as Starling resistors^5^. The BCV transmural pressure is increased by 327% in hydrocephalic dogs^5^. The cause of this has never been satisfactorily explained by the Starling resistor mechanism. This suggests that the Starling resistor model is incomplete in canine hydrocephalus. Such a large increase in transmural pressure should be visible as an increase in BCV size (see Fig. 1e,f).

In a recent paper studying children with hydrocephalus, in those between 4–15 years of age there were mild stenoses of the sagittal sinus, the transverse sinuses and the sigmoid sinuses of 35%, 30% and 41% respectively compared to controls^7^. These stenoses should mildly increase the sinus pressures, similar to the findings in the dogs as already discussed.The purpose of the current study is to (1) estimate the superficial and deep venous territory pressure difference based on blood flow and collateral flow in children and (2) to measure the cross-sectional area of the BCVs at the superficial sinus junction and vein of Galen at the deep junction to see if the predicted pressure difference resides within the BCVs.

## Results

The mean blood flow and sinus size findings are summarized in Table 1. The raw data is appended online in supplementary table S1.

**Table 1:** Blood flow and vein size.

	Age Years	Control	Blood flow					
		Arterial inflow ml/min	SSS outflow ml/min	ST outflow ml/min	SSS % return%	ST % returnml/min		
Mean	9.2	996	558	162	56.5	16.2		
SD	4.1	216	132	60	8.2	4.3		
n	22							

		Control	Vein size					
							BCV area mm^2^	Galen area mm^2^
Mean	9.6						8.5	11.9
SD	3.3						2.7	4.9
n	72							

		Hydrocephalus						
Mean	9.6	1056	474	144	43.6	14.0	10.4	11.2
SD	3.3	336	240	42	15.1	3.5	3.3	3.9
n	18							
MWU	0.76	0.70	0.06	0.34	0.003*	0.09	0.0008*	0.60

*BCV* bridging cortical vein, *ml/min* milliliters per minute, *mm2* millimeters squared, *MWU* Mann–Whitney U test, *SD* standard deviation, *SSS* superior sagittal sinus, *ST* straight sinus; *significance < 0.05.

In the controls, as expected there were moderate to strong positive correlations between arterial inflow and both sagittal sinus (r = 0.73, p = 0.0001) and straight sinus outflow (r = 0.68, p = 0.0009). All other correlations were weak or nonexistent.

**Figure 2:**
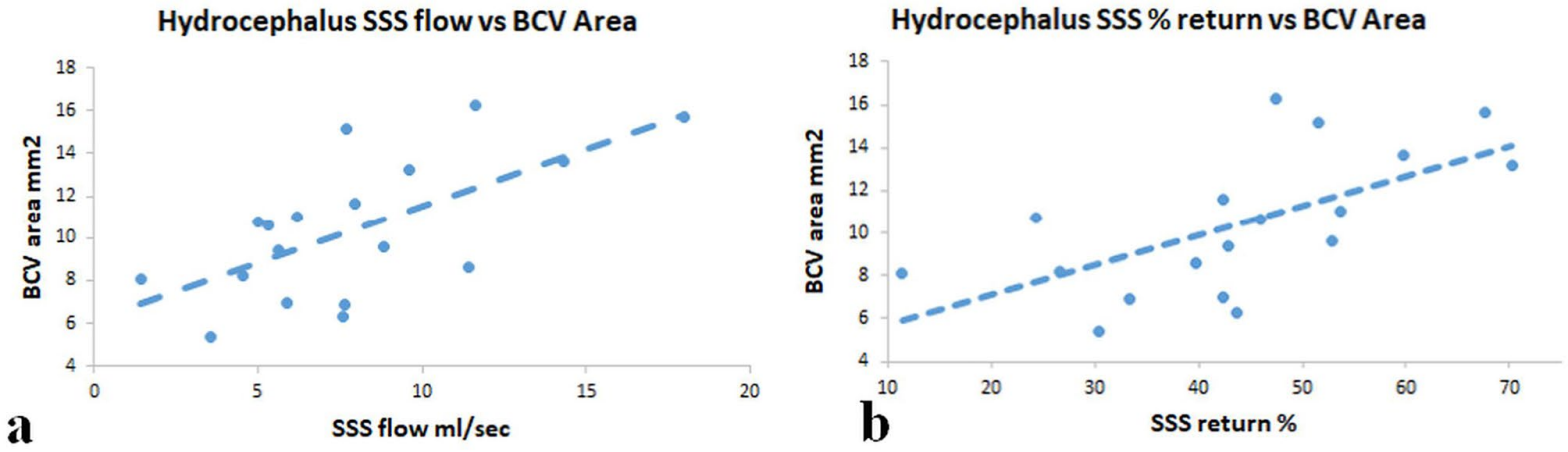
Scatter plots of significant correlations. (**a**) A scatter plot of hydrocephalus superior sinus flow vs bridging cortical vein area showing a positive correlation (r = 0.65, p = 0.004). (**b**) A scatter plot of hydrocephalus superior sagittal sinus percent return vs bridging cortical vein area showing a positive correlation (r = 0.67, p = 0.002).

In the hydrocephalus patients, there was no significant difference between communicating or non-communicating or between active and compensated hydrocephalus for any metric. There was no significant correlation between the ventricular size or the aqueduct flow rate for the communicating hydrocephalus patients with any other metric acquired. Comparing hydrocephalus with controls, the sagittal sinus venous return was reduced by 12.9% (p = 0.003). Comparing the 288 control BCVs with the 72 hydrocephalic patient BCVs sampled, showed the mean bridging cortical vein area was increased by 22% (p = 0.0008). Similar to the controls, there were moderate to strong correlations between the arterial inflow and the sagittal sinus (r = 0.67, p = 0.002) and straight sinus outflow (r = 0.76, p = 0.0002). There was a moderate negative correlation between arterial inflow and straight sinus percentage return (r = − 0.64, p = 0.004) but not the sagittal sinus percentage return. There was a moderate positive correlation between the sagittal sinus flow and the bridging cortical vein area (r = 0.65, p = 0.004) (see Fig. 2a) and between the sagittal sinus percentage return and bridging vein area (r = 0.67, p = 0.002) (see Fig. 2b).

### Modelling

In human cadavers the mean diameter of the bridging veins entering the sinus at the vertex is 3.1 mm and at angiography at the same site it is 3.3 mm^8^. Which gives a stress free radius (cadavers) of 1.55 mm and a stress free area of 7.55 mm^2^. The area with a normal transmural pressure (angiography) would be 8.55 mm^2^. The mean thickness of the human bridging veins is 0.044 mm^9^. The normal cortical vein transmural pressure for primates is 2.8 mmHg^10^. Placing these values into Eq. (1) gives a circumferential Young’s modulus of 0.163 MPa. Given the normal stressed state area of the BCV’s from the present study is 8.5 mm^2^ and the above parameters for wall thickness and elastic modulus, we can derive the stress free radius for the present study to be 1.55 mm from Eq. (1). Given the stressed state BCV area for the hydrocephalus patients is 10.4 mm^2^ in this study, the transmural pressure from Eq. (1) would be 6.9 mmHg or 4.1 mmHg higher than normal.

## Discussion

The hypothesis made in this paper is that the pressure difference required to drive ventricular enlargement in hydrocephalus resides predominately within the bridging cortical veins. In humans, on average 18 cortical veins drain directly into the sagittal sinus without lateral lacunae^11^. The bridging vein diameter remains constant along its subarachnoid course but dilates at a short section called the outflow cuff segment as it joins the sinus^11^. The collagen fibres are helical at this junction and longitudinal elsewhere^11^. In humans the bridging cortical veins are suggested to be passive Starling resistors as previously described. Piechnik et al. predicted that the trans-mural cortical venous pressure change from hyperemia to severe ischemia should only be in the order of 1.5 mmHg, with the physiological range being considerably less^12^. This appears to be incorrect. In normal dogs, the cortical vein transmural pressure is 1.6 mmHg. This increases by 12.5% when mock CSF is infused, by 63% from inhalation of carbon dioxide and by 81% by jugular vein compression, with all these changes giving an increase in ICP of approximately 10 mmHg^5^. In hydrocephalic dogs the ICP rises by 4.9 mmHg but the transmural pressure increases by 327%. In human subjects, the bridging veins show a mean diameter of 2.04 mm under normal intracranial pressure and increase to 2.65 mm under an increased ICP^13^, a 69% increase in cross-sectional area. Thus, the purpose of the current study is to investigate whether BCV dilatation indicates a pressure gradient exists between the venous territories in childhood hydrocephalus.

In this study we have used changes in blood flow to estimate the magnitude of the pressure difference which should exist within the venous system. It has been found that an elevation in venous pressure from whatever cause, directs a larger percentage of the arterial inflow to exit via the smaller venous channels through the scalp, face and over the brain convexity through the veins of Trolard and Labbe as collateral flow. This collateral flow reduces the percentage of the arterial inflow returning via the main pathways in children at risk of idiopathic intracranial hypertension (IIH) by 11% in the SSS and 4% in the straight sinus^14^. This represents a collateral flow bypassing the main sinuses of 146 ml/min and 53 ml/min respectively. This indicates an increase in pressure both within the superficial and deep venous territories. Children with IIH require a minimum elevation in ICP of 3.8 mmHg above normal to be symptomatic, with this all originating from causes below the Torcular^15^. Therefore, we can equate the 146 ml/min reduction in sagittal sinus venous return in IIH with an estimated minimum 4 mmHg increase in sagittal sinus pressure. In the current study the superior sagittal sinus percentage venous return was reduced by 12.9% or 136 ml/min, suggesting a similar 4 mmHg increase in venous pressure. Unlike IIH, the percentage venous return in the straight sinus deep venous territory was unchanged in hydrocephalus, suggesting the pressure change arises above the Torcular within the superficial venous territory and not the deep territory. Aso et al. using a different MRI technique came to the same conclusion, suggesting that ventriculomegally is correlated with a relative increase to the resistance to blood flow in the superficial venous system of the brain compared to the preserved drainage in the deep system^16^.

In a study into children with hydrocephalus, there was a 35% effective area stenosis of the sagittal sinus^7^. In a modelling study, a 38% reduction in the sagittal sinus area in hydrocephalus lead to a 0.7 mmHg increase in venous pressure^1^. So the effect of the stenosis in the sagittal sinus in children is probably less than 1 mmHg. This leaves a minimum 3 mmHg of pressure elevation elsewhere in the venous system to account for the collateral flow in hydrocephalus in children. Therefore, the blood flow data suggests a pressure increase in the BCVs of the sagittal sinus of a minimum of 3 mmHg but no increase in the vein of Galen.

In hydrocephalus, the positive correlation between the sagittal sinus outflow volume and BCV area in hydrocephalus indicates that blood flow affects the transmural pressure similar to that in dogs made hyperaemic by elevating the inspired carbon dioxide level (see Fig. 2a). In hydrocephalus there was a positive correlation between the size of the BCVs and the superior sagittal sinus percentage return (Fig. 2b), suggesting that in those individuals who have a poorer availability of collateral pathways, there are higher transmural BCV pressures. In this study there was an average 22% increase in BCV area in hydrocephalus compared to controls. The modelling performed suggests a 4.1 mmHg increase in transmural pressure to bring this about. This modelling was based on a study comparing cadaver BCV vein size to angiography^8^ which was used to estimate the circumferential Young’s modulus. This was necessary because the circumferential modulus has not been measured in humans. The longitudinal BCV modulus, however, has been measured and found to be 30.7 MPa in one study^17^ and 25.7 MPa in another^9^. The orientation of the collagen fibres in the body of the BCVs is longitudinal^11^ indicating the longitudinal modulus is likely to be much higher than the circumferential one. In pigs, there is a linear response of the diameter of the bridging veins to the transmural pressure. At zero transmural pressure the mean diameter was 0.90 mm and at 8 mmHg it was 1.07 mm with a mean wall thickness of 0.03 mm^18^. Thus, the circumferential modulus in pigs can be estimated using Eq. (1) to be 0.08 MPa compared to our value in humans of 0.163 MPa and both compare to the 0.4 MPa found in silicone tubes^19^.

The bridging vein for the deep system is the vein of Galen. It was found to average 11.2 mm^2^ in controls in the present study which compares to a study using MRV measurement where the average vein of Galen was 4.04 mm in diameter equivalent to 12.8 mm^2^ in area^20^. There was no difference between the hydrocephalus patients and controls indicating likely no increase in transmural pressure.

**Figure 3:**
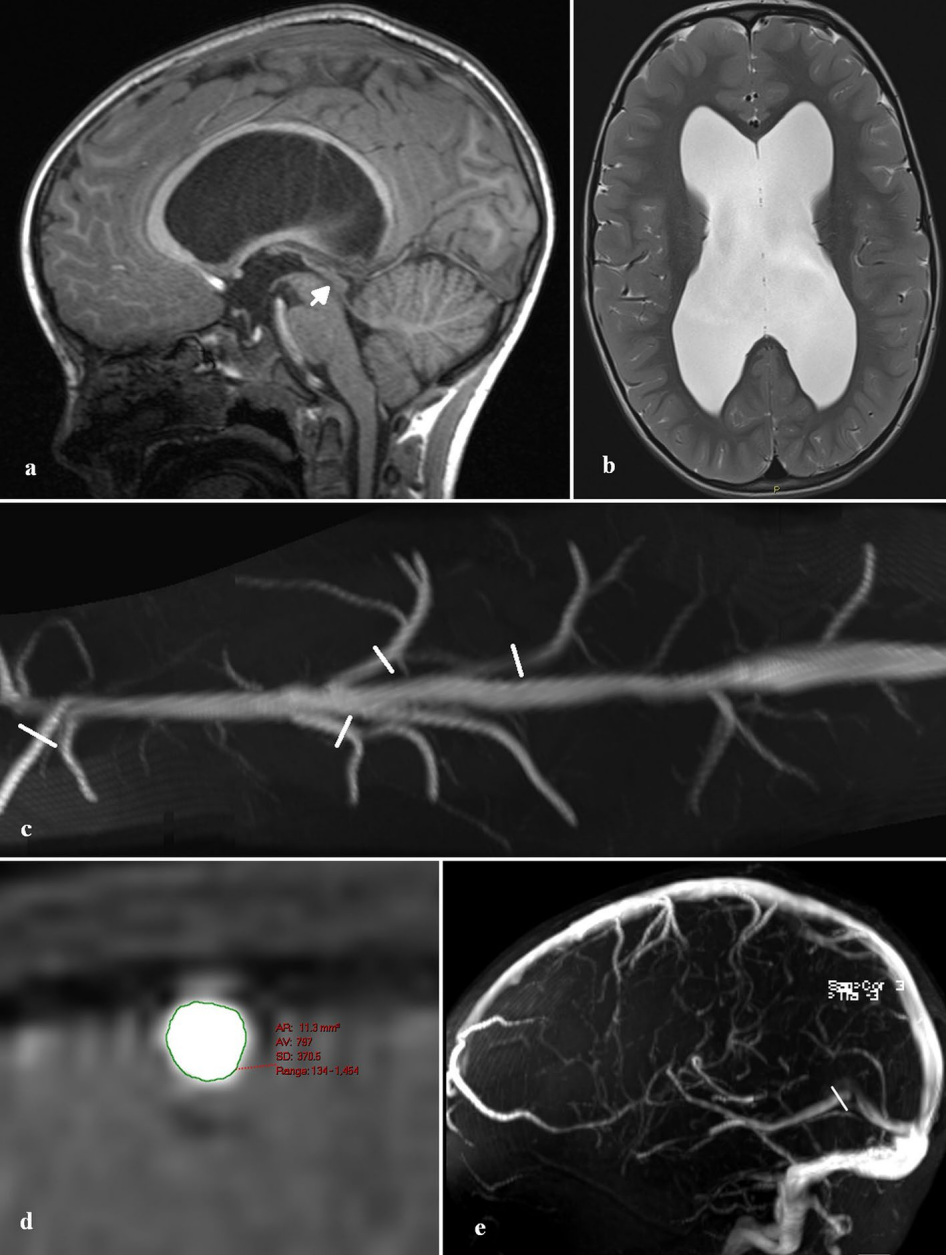
Site of vein measurements in a child with obstructed hydrocephalus. (**a**) sagittal T1 image of patient 2 showing the obstructed aqueduct of Silvius (arrow). (**b**) Axial T2 image showing the ventricular dilatation. (**c**) Curved maximum intensity projection image obtained from the MRV data orientated along the course of the superior sagittal sinus (outflow towards the right) showing the site and orientation of the BCV measurements (white lines). (**d**) MRV reconstruction of a cortical vein showing an area measurement. (**e**) MRV reconstruction showing the site of the vein of Galen measurement (white line).

The passive Starling resistor model of the BCVs is incomplete. There appears to be no anatomical reason a transmural pressure significantly higher than normal can be maintained by the venous system. In the current study all of the cortical veins in the hydrocephalus patients appeared fully open all the way to the sagittal sinus. Figure 3c is an example from patient 2 who had obstructive hydrocephalus. Given there is no structural reason for an increase in transmural pressure, the question arises, could there be a functional cause?

Mahat et al. noted, vessels of differing elastic properties such as in the cardiovascular system may exhibit the characteristics of an impedance pump^21^. Hickerson et al. define an impedance pump as a device which uses a mismatch in impedance between two adjacent tubes to drive flow without valves^22^. By compressing the more compliant tube periodically in a position adjacent to the less compliant, pressure waves are set up which reflect at the points of impedance mismatch. The constructive interference of these waves will generate a net flow. The flow rate is non-linearly dependant on the compression frequency and is pulsatile. Hickerson et al. define 4 criteria to indicate if an impedance pump is operating; (1) a difference in impedance between the tubes, (2) distal compression point in the compliant tube, (3) maximal flow occurs at the resonant frequency and (4) flow exiting the tube is typically pulsatile. Clearly, there is an impedance mismatch between the cortical veins and the sagittal sinus satisfying criteria (1). With regards to criteria (2) and the site of BCV compression. The peak pulse in the cortical veins lags behind the sinuses, indicating that the pulse exits the arterial tree passing into the subarachnoid space and re-enters the cortical vessels just before their junction with the sinuses^23^ i.e. at the cuff region of the vein. In the deep system there is evidence of compression of the distal vein of Galen as it joins the straight sinus^24^. In criteria (3), maximum efficiency occurs at the resonant frequency of the system. In dogs, the intracranial system selectively absorbs the intracranial pulse pressure waves at the resonant cardiac frequency^25^. Similarly, the normal human intracranial system selectively absorbs the CSF pulse pressure at a resonant frequency around the heart rate i.e.1.49–2.05 Hz (89–123 beats per minute)^26^. Finally, using criteria (4), the flow exiting the cortical veins is pulsatile. Measurement of the bridging vein flow upstream from the compression site in normal patients gave a pulsatility index (P.I.) of 0.42^27^. Therefore, we suggest the bridging cortical veins fulfil all of Hickerson et al.’s. criteria for being an impedance pump. How would this materially affect the transmural pressure? The highest flow rate in a 2 mm silicone impedance pump was 16 ml/min^19^. From our study, the sagittal sinus flow rate is 558 mls/min and there are on average 18 cortical veins providing this flow^11^, therefore each cortical vein has an average flow of 31 mls/min. Therefore, impedance pumping could significantly reduce the BCV pressure if operating efficiently. Indeed, the geometry of the cortical veins may be optimised to improve pumping efficiency. It has been shown that placing a loop in an impedance pump increases the average flow rate by between 31–72%^28^. The BCVs loop backwards and empty against the SSS flow with the average outflow angle being 35°^8^. Similarly, placing a dilatation at the site of the tube compression (increasing the diameter by 20% compared to a straight tube) can increase the average flow by fourfold^29^. The outflow cuff as previously described is such a dilatation.

If an efficiently performing BCV impedance pump significantly reduces the transmural pressure in normal individuals we could hypothesise that failure of such a mechanism in hydrocephalus could account for the elevated transmural pressures found. In hydrocephalic dogs, the pulsation absorber mechanism appears lost^30^. In hydrocephalic dogs there was a 53% increase in ventricular pulse pressure and an 85% increase in cortical vein pulse pressure but the sagittal sinus pulse pressure was not significantly changed compared to controls^31^, indicating pulsation absorber failure.

In human hydrocephalus, the resonant pulsation absorber mechanism is also lost^26^. In the intracranial cavity the pulsation absorption consists of two components, the CSF pulsating backwards and forwards in the spinal canal and the venous compression^23^. In an impedance pump consisting of two compression regions in differing locations maximal flow will occur at a fixed phase lag between the two regions of compression. In an experimental system consisting of two compression regions, a fixed phase lag of 55° gave the maximal flow rate. Reducing the phase lag to zero reduced the flow rate tenfold^32^. In humans the phase lag from the artery expansion to the spinal canal at C2 level is + 5.1 ± 10.5°^33^. The phase lag in the sagittal sinus is –50° after the arterial pulse^27^, giving a phase difference of 55° between the spinal canal pulsation and the cortical vein compression. In hydrocephalus of middle age, this lag is reduced by 58%^34^ and by 51% in elderly NPH patients^35^. The shift occurs because the craniospinal compliance is reduced in hydrocephalus with a significantly reduced amount of CSF expelled from the foramen magnum despite the increased CSF pulse pressure^36^. The alteration in phase associated with hydrocephalus would decrease the phase difference from 55° to 23° and significantly reduce the pumping efficiency.

In addition, increasing the outflow resistance in an impedance pump model reduced the peak flow rate because the tube was made stiffer^22^. Note, in dogs, increasing the outflow resistance by jugular vein compression increased the transmural venous pressure by 81%^5^. Similarly, increasing the outflow pressure by increasing the transmural pressure within the tube in an experimental impedance pump from 2.8 mmHg to 6.6 mmHg reduced the peak flow to zero^22^. These pressures are almost identical to the ones modelled in the normal and hydrocephalic children in this study. Suggesting a complete failure of impedance pumping in children may exist. This sets up a positive feedback loop, i.e. an increase in BCV transmural pressure will both be the result of, and also contribute to, the failure of the impedance pumping. Such a positive feedback loop would be expected to be unstable over short time periods and lead to variations in ICP over time. These pressure waves are known as B waves and are a feature of the ICP in hydrocephalus^37^. Occurring in both communicating and obstructive hydrocephalus^38^. In human hydrocephalus, the BCV outflow impedance appears to increase, with the upstream pulsatility index being 0.28 (compared to 0.42 in controls). This rebounds to 0.8 once the intracranial compliance is increased by intraventricular shunt placement. The straight sinus pulsatility was unchanged from normal both before and after shunting^39^, suggesting it is unaffected.

One may ask, do the present findings have any clinical relevance with regards to the treatment of hydrocephalus in children? We think there are several areas where the findings have clinical relevance; (1) current treatment is only aimed at improving the CSF flow and not optimised for improving the venous pressure, (2) obstructed hydrocephalus may need to be treated similarly to communicating hydrocephalus (3) the findings may have relevance to adult hydrocephalus.

The classical model of hydrocephalus stresses only an increase in the outflow resistance of CSF flow at either the aqueduct or the arachnoid granulations with an accumulation of the fluid behind the obstruction. Thus, current treatments are targeted to reducing the resistance to CSF flow and not altering the impedance of the vascular outflow. This may suggest a need to modify shunt treatment. Although the treatment of communicating hydrocephalus vastly improved with the introduction of the differential pressure valve, it can be argued that little further improvement has occurred since^40^. Shunt over drainage is an ongoing problem in children despite the introduction of anti-syphoning valves^40^. Bergsneider suggests the problem likely rests with our incomplete, overly simplistic understanding of the hydrodynamics. The next generation of shunts or other treatments for hydrocephalus will need a better understanding of the interrelation between the pulsatile cerebral perfusion and CSF dynamics^40^. The major contributor to over drainage is gravity dependent siphoning in the upright position, for which anti-siphon devices have been devised. However, increased intracranial pulsations can also lead to over drainage with the CSF flow being proportional to the pulse amplitude^41^. Thus the present study would suggest that modifications to either the shunt apparatus to increase its compliance and/or a craniectomy ± duroplasty to increase the intracranial capacitance may be necessary to dampen and alter the phase shift of the pulsations. A recent paper indicates posterior fossa decompression successfully ameliorates the hydrocephalus associated with Chiari I malformation in 90% of cases without further treatment^42^. Thus, this therapy could improve the venous outflow drainage as well as make the CSF absorption more physiological.

With regards to treating obstructed hydrocephalus, the current study suggests there is little difference between obstructed and communicating forms. Even in successfully treated aqueduct obstruction, children appear to have supranormal CSF volumes in the long term^43^. This contrasts with shunted patients, who continue to exhibit declining ventricular volumes after 6 months^43^. The observation that the final volumes are much higher than normal implies that the absorptive mechanism works less well in these patients in comparison to normal subjects and it thus appears that successful ventriculostomy produces a state of compensated communicating hydrocephalus^43^. In a study of children where a third ventriculostomy was performed to treat obstruction, and the stoma was judged to be patent, the mean stroke volume across the stoma was 185 µL^44^ compared to the normal aqueduct stroke volume for children of 15 µL^45^. Such a hyperdynamic flow is similar to that found in the aqueduct in communicating hydrocephalus. Therefore, if symptoms persist, progress to shunt insertion should not be delayed.

In adults, with longstanding obstructed hydrocephalus, the overall reduction in compliance of the system has been shown to be unchanged post third ventriculostomy^46^ and is identical to the reduction in intracranial compliance found in communicating hydrocephalus^34^. In adults, there is a reduction in the percentage of the arterial inflow returning in the SSS but not the straight sinus in both forms of hydrocephalus^34^, similar to the current study’s findings in children,. Thus, the present study may have some utility in understanding adult hydrocephalus as well.

Hydrocephalus tends to be initially diagnosed either by head ultrasound in the infantile period or by CT scanning later. The MRI studies reviewed in this paper were requested by the referring neurologist or neurosurgeon at their discretion and thus there is a possible selection bias with those patients deemed to be at greater risk i.e. either symptomatic or with larger ventricles being over represented in this study. Despite this, there was no appreciable correlation between the metrics used and either the patients symptoms or the ventricular size.

## Methods

### Subjects

In a previous study, the radiology information system at a tertiary referral hospital was retrospectively interrogated to retrieve all data from children between birth and 15 years of age who had an MRI with MRV and flow quantification, for the investigation of any form of hydrocephalus between January 2009 and January 2019^7^. Eighteen children between 4 and 15 years were enrolled from this study into the current study. The selection criteria for the original study were a treatment naive hydrocephalus not secondary to tumor. The ventricular size index was measured on axial T2 images as the size of the frontal lateral ventricles compared to the size of the frontal lobes and a figure of 0.3 was used as a cut-off. Obstructive hydrocephalus was diagnosed if there was no flow through the aqueduct of Silvius and communicating hydrocephalus if the aqueduct stroke volume was above 40 µl per systole on dedicated flow quantification imaging. There were 7 with obstructed hydrocephalus with no flow and 11 with communicating hydrocephalus with an average aqueduct flow of 201 ± 215 µl. There were 8 females and 10 males. The pediatric neurologist (GMS) designated the patients as active or compensated hydrocephalus based on a chart review. Further information about the ventricular size and clinical findings is available in the previous study^7^. Seventy two control MR venogram (MRV) patients were enrolled from the previous study^7^. They had an MRI study for indications not related to headaches, large head, raised intracranial pressure or hydrocephalus, in which the subsequent MRI was found to be normal. There were 40 males and 32 females. There were 22 control patients with MR flow quantification studies reenrolled from the previous study with 10 males and 12 females.

### Ethics approval and consent to participate

Informed consent was obtained from all patients and parents enrolled in this study. The study was approved by the Hospital ethics committee, therefore, the study has been performed in accordance with the ethical standards laid down in the 1964 Declaration of Helsinki. The authorization number 2019/ETH12487 was issued.

### MR and analysis

All patients were imaged on a 3.0 T superconducting magnet (Avanto; Seimens, Erlangen Germany). In all patients, a standard brain MRI consisting of 3DT1 sagittal, T2 axial, FLAIR axial and diffusion weighted axial images was performed. An MR phase contrast flow quantification sequence was acquired with retrospective cardiac gating. The TR was 26.5 ms, TE 6.9 ms, flip angle 15º, slice thickness 5 mm, matrix 192 × 512, FOV 150 and a single excitation. The first velocity encoding value was 150 cm/s with the plane set to pass through the skull base, to cross the carotid and basilar arteries. A second acquisition had a velocity encoding value of 40 cm/s and was angled to measure the mid portion of the straight sinus and the sagittal sinus approximately 3 cm above the Torcular as per a previous study^14^. A time of flight MRV acquisition was performed in the off sagittal plane. The MRI imaging was sourced from the hospital picture archiving and communication system (PACS) and therefore all measurements were performed on the original data.

Areas of interest were placed around the carotid and basilar arteries for all patients, to give the total arterial inflow at the skull base by summing the individual flows. The arterial inflow data has been previously published^7^. Regions of interest were placed around the straight sinus and superior sagittal sinus to give the two venous sinus outflows. This data has not been previously published. Background subtraction was used to remove the effect of eddy currents in all results. The percentage of the arterial inflow drained by both the sagittal sinus and the straight sinus was calculated for each patient. The MRV data was reformatted to display the length of the sagittal sinus (see Fig. 3). The two largest BCVs on the right and left sides were selected and reconstructions performed perpendicular to their longitudinal axis 1 cm from their junction with the sinus (see Fig. 3c). The cross-sectional area was measured (see Fig. 3d). The four veins were then averaged for each patient. The vein of Galen was measured 5 mm from its junction with the straight sinus perpendicular to its longitudinal axis (see Fig. 3e). Mean and standard deviations were obtained for each group. A Shapiro–Wilk Test was used to test for normality of the data. Differences between groups were tested using a Mann–Whitney U test. Correlation amongst variables was performed using a Spearman’s Rho test. An α ≤ 0.05 was used to assess statistical significance for all tests.

Mathematical modelling to estimate the transmural pressure was performed. The equation relating the transmural pressure to the cross-sectional area of a vessel is

1 $$Ptm=\frac{4Eh}{3Ro}(1-\sqrt{\frac{Ao}{A}} )$$

where Ptm is the transmural pressure, E is the circumferential Young’s modulus of the wall, h is the wall thickness, Ro is the radius in the stress free state, Ao is the area in the stress free state and A is the area following the applied transmural pressure^32^.

## Supplementary Information

Supplementary Information.

## Data Availability

All data generated or analysed during this study are included in this published article (and its Supplementary Information file).
